# New evidence for Early Pleistocene use of fire at Wonderwerk Cave (South Africa)

**DOI:** 10.1371/journal.pone.0347480

**Published:** 2026-06-01

**Authors:** M. Dolores Marin-Monfort, Candice L. Shaw, Filipe Natalio, Liron Grossman, Peter Andrews, Joaquín Campos, Sara García-Morato, José M. Pereira, Alicia Pons, Michael Chazan, Liora Kolska Horwitz, Yolanda Fernández-Jalvo

**Affiliations:** 1 Department of Geology, Universidad Nacional del Sur - Consejo Nacional de Investigaciones Científicas y Técnicas (UNS-CONICET), Bahía Blanca, Argentina; 2 Department of Earth Sciences, University of Toronto, Toronto, Ontario, Canada; 3 Department of Chemistry, NOVA-FCT, University of Lisbon, Lisbon, Portugal; 4 Department of Plant and Environmental Sciences, Weizmann Institute of Science, Rehovot, Israel; 5 Department of Earth Sciences, The Natural History Museum, London, United Kingdom; 6 Instituto de Óptica Daza de Valdés, Consejo Superior de Investigaciones Científicas (CSIC), Madrid, Spain; 7 Institute of History, Consejo Superior de Investigaciones Científicas (CSIC), Madrid, Spain; 8 Museo Nacional de Ciencias Naturales, Consejo Superior de Investigaciones Científicas (CSIC), Madrid, Spain; 9 JPereira.Net, Digital Heritage, Guadalajara, Spain; 10 Evolutionary Studies Institute, University of the Witwatersrand, Johannesburg, South Africa; 11 National Natural History Collections, The Hebrew University, Jerusalem, Israel; Universidad Autonoma de Madrid, SPAIN

## Abstract

Tracing the earliest evidence of burning in archaeological contexts is essential for understanding the emergence of fire use—an innovation that underpinned critical behavioral and biological developments in the genus *Homo*. However, identifying unambiguous traces of early fire use remains challenging. To enhance detection of incipient burning in early occupation layers, we introduce a rapid, non-invasive protocol based on bone luminescence properties, validated through comparison with Fourier Transform Infrared spectroscopy (FTIR). Using these methods, we provide evidence for fire use in two Early Pleistocene (Acheulean) deposits at Wonderwerk Cave (South Africa), extending the chronology of one of the world’s earliest paleo-fire records. This combined approach improves the resolution with which early fire use can be identified and opens new avenues for investigating the emergence of pyrotechnology in deep time.

## Introduction

The use of fire is a critical component of the evolutionary dynamics of genus *Homo* that led to a momentous shift in the relations between hominins and their natural and cultural environments. The tempo and scale of innovation in hominin pyrotechnology are the subject of debate, yet it seems likely that early *Homo* was only able to acquire fire from natural, mostly seasonal wildfire resources, bring it to their occupation sites, and maintain it until it was extinguished. The ability to make and control fire only developed much later [1,2].

Currently, the oldest evidence of fire use associated with hominin activity (though not necessarily control of fire) comes from sites in Africa. Early hominins in Africa were associated with a tropical savanna biome that today is responsible for ~60% or more of global carbon derived from biomass burning, resulting in the sobriquet “fire continent” for Africa [3,4]. Thus, natural fires on this continent would have been ubiquitous. Fire-cleared grasslands may have allowed early hominins to detect predators more efficiently, as observed in current primate populations [5,6], and also facilitated spotting of potential prey [7]. Fire use by hominins also provided warmth and extended daylight hours, protected against predators or scavengers, and facilitated consumption of a wide range of meat and plant foods through exposure to flames. In sum, together with lithics, fire was, a decisive innovation enabling hominins to be independent and modify many crucial facets of their natural and cultural environments.

The most robust dataset supporting early fire use currently derives from the Acheulean of Wonderwerk Cave (South Africa), Excavation 1, Stratum 10 (hereafter St. 10), dated to ~1.0 Ma. This is based on finds of burnt bone, stone, sediment, and *in situ* ash together in the same layer [8]. Additionally, in Member 3 at Swartkrans Cave (South Africa, dated to ~1.0–1.5 Ma) [9], burnt fossil bones have also been reported, but the deposit appears to be in a secondary context [10]. Evidence for burnt lithics and/or sediment has been documented in several Early and Middle Pleistocene open-air sites in Africa [11–13] such as Koobi Fora (Kenya, ~ 1.5 Ma) [11,14] However, natural wildfire rather than hominins as the source of burning in these African open-air localities, becomes a factor not encountered inside deep caves such as Wonderwerk Cave. Moreover, while it has been suggested that burnt sediments in many open-air localities may have eroded away and so are not preserved [15,16], at Wonderwerk Cave no evidence of sediment transport has been detected above basal strata that could result in secondary deposition of burnt materials [17,18].

Gowlett et al. [19] have argued that evidence for early hominin use of fire is best supported by the association of lithic artefacts, butchered bones and evidence for repeated episodes of burning in the same context. However, in general, unequivocal evidence for clearly defined hearths is absent in early sites associated with *H. erectus*, possibly as the result of an opportunistic mode of engagement with fire.

The importance of discovering the earliest uses of fire, such as fire introduced by hominins to their occupation sites, is basic to the identification of the much later domestication of fire associated with the regular incorporation of cooked food into the hominin diet [20,21]. This innovation of fire use increased caloric return made available from cooked meat, reduced energetic consumption in chewing and digestion, facilitating greater investment in brain development [22–24] leading to a significant increase in neocortex size, in the hominin lineage [25]. The integral role of fire in hominin adaptations may also have facilitated hominin dispersal (initiated by *Homo erectus*) out of Africa and also had implications for the co-evolution of social structure and communication [24–26]. The earliest evidence for control and domestication of fire has been identified outside of Africa, in Gesher Benot Ya’aqov in Israel (dated to ~0.8 Ma). In this site, repeated use of the same combustion areas over time described by the research team as indicating hearths [27,28].

Detecting signs of hominin involvement in fire use is, therefore, a highlight in hominin evolution. The suite of existing methods to detect fire-use in fossil sites include micromorphology to identify charcoal, ash, and color change to detect burnt sediments, surface and internal alterations to lithic material, and detection of burnt bones based on their color and taphonomic surface modifications [11,15,28–33]. Color classification of bone is frequently used in archaeology and forensic studies as a first approach. Sampling by color is a useful criterion to separate out potentially burnt bones on which to then apply other methods, but cannot be considered evidence of fire by itself alone. Color may be misleading due to diagenetic changes in the bone such as manganese (Mn) staining, (which may mimic the black charred bones) or fluoridation which may be mistaken for calcined white bone. Furthermore, color perception may be subjective as restricted by the chromatic capacity of the observer [34]. In our former taphonomic papers, we addressed these challenges by employing different spectrometric methods to bones, i.e., Raman spectrometry, scanning electron images (SEM), energy dispersal spectra (EDS) and cathodoluminiscence (CL) [35,36]. At Wonderwerk Cave, fire associated with an early Acheulean activity (St. 10) was previously documented using FTIR and micromorphology [8]. Burnt bones were also identified using SEM/BSE-EDS spectrometry [37]. This methodology (see also methods) was applied to small mammal fossil assemblages of Wonderwerk [35]) and other sites [38]. Currently, Fourier Transform Infrared Spectroscopy (FTIR) has become the most widely applied method to detect thermal modifications in lithics, sediments and bones [8,14,37,39–42]. However, FTIR too has limitations since it cannot unequivocally document burning below the temperature threshold of ~ 537 ºC [40]. Since char does not maintain its molecular structure past 537 ºC in an oxidizing environment, this is a significant limitation, suggesting that the frequency of burning of black bones registered by FTIR is likely to underestimate the true extent of burning [39]. It is thus apparent that investigations to identify the modality of early hominin engagement with fire, particularly the spatial structure of burning within archaeological sites require the development of additional robust methods. The luminescence method presented here shares the limitations of FTIR and furthermore cannot be applied to black bones. However, luminescence is complementary to FTIR as it is independent and, unlike FTIR, can be applied to large samples as it is non-destructive and rapid.

In order to enhance and facilitate the detection of early stages of burning, we introduce here a rapid, non-invasive protocol based on the luminescence properties of bones (i.e., emission of light at a different wavelength than the incident light). We have again used color classification as a first step in separating out potentially burnt from unburnt bones, specifically applied here to grey and white colored bones to test for bone luminescence. We validated this approach through systematic comparison with FTIR analysis applied to the same fossils that yielded luminescence. Results presented here provide conclusive evidence and confirm burning associated with hominins in St. 10 and St. 11, the later constrained to 1.79–1.07 Ma (see discussion below). Fire in St. 11 pushes back the age of the earliest evidence for fire and indicates that fire was repeatedly brought by hominins into the interior of Wonderwerk Cave [35].

## Wonderwerk Cave

Wonderwerk Cave (27° 50′ 44.22″ S, 023° 33′ 14.00″ E) is located 60 km south of the town of Kuruman (Northern Cape Province). The site has yielded a fossil and archaeological record covering almost 2 million years of human occupation [43–45], best preserved in Excavation 1. As noted above, unlike the roughly contemporaneous deposits in South African hominin sites in the Cradle of Humankind (particularly Swartkrans and Sterkfontein), Wonderwerk Cave has yielded a stratigraphic sequence of *in situ* deposition rather than secondary infill, and there is no mechanism other than hominin action that can account for the accumulation of lithic artefacts in the contexts where they have been recovered [8,17,37,43–45]. Both taphonomic and micromorphological/stratigraphic analyses of the Wonderwerk Cave sequence show no evidence of post-depositional transport of faunal or lithic remains [17,18].

**Fig 1:**
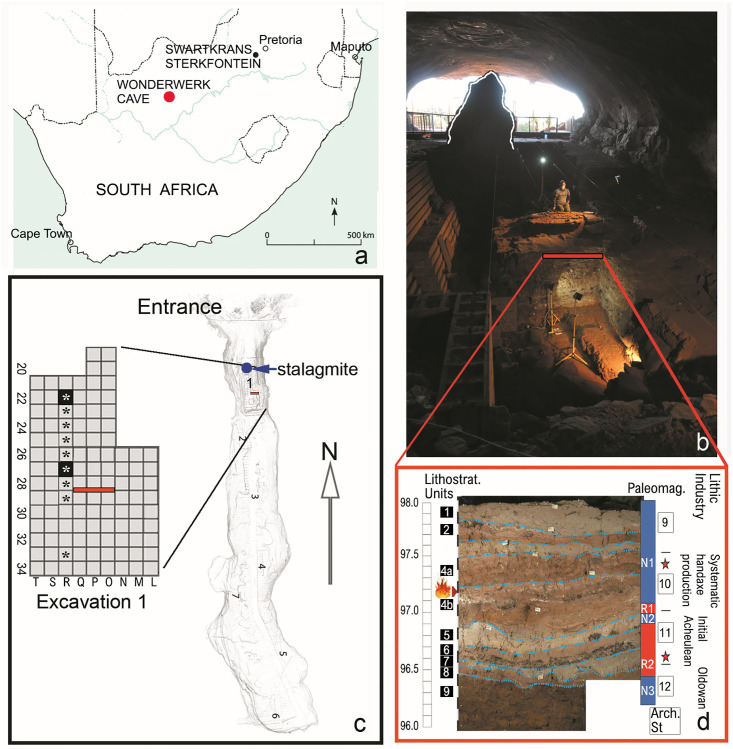
(a) Location of Wonderwerk Cave (red dot) in South Africa. **(b)** Excavation 1 area showing the stalagmite landmark (outlined in white) and the section (red line) displayed in (d). **(c)** Plan of the excavation grid (yard^2^), R22 and R27 (in black) show intense evidence of cremation of small mammal remains (asterisks indicate the fossils analyzed here by FTIR + luminescence; see Fig 4 and the text). Background image for figures b-c based on data collected by the Zamani team, copyright Wonderwerk Cave Research Project/Michael Chazan. **(d)** North profile showing the lithostratigraphic units (Lithostrat. Units 1–9), paleomagnetic zones (Paleomag.: N: normal or R: reverse), archaeological strata (Arch. St. 12 to St. 9), and associated lithic industries. The emoji of fire on the left vertical axis shows the location of samples in St. 10 that attest to burning in the cave ca. 1Ma by Berna et al., 2012. Background image for figure d, copyright Wonderwerk Cave Research Project/Michael Chazan. The red stars on the right vertical axis denote the two strata (St. 10 and St. 11) analyzed here. Note that the two strata analyzed here are separated by ca. 80 cm of deposits comprising three lithostratigraphic units.

Excavation 1 in Wonderwerk Cave produced a sequence of seven Earlier Stone Age strata (St. 6−12; Fig 1) that were identified by Peter Beaumont during excavations undertaken between 1978 and 1996 [46]. Beaumont’s strata attest to the evolution of stone tool industries, with a small-tool dominated assemblage attributed to the Oldowan in St. 12, at the base of the sequence [47]. This is followed by the first appearance of rough handaxes in the lower part of the overlying St. 11, consistent with the onset of the Acheulean, while St. 10−9 which lie above, there is further development in the regularity of handaxe production, with a shift towards even more refined handaxes in overlying Strata 8−6 [47].

Geological analysis carried out subsequent to Beaumont’s excavations, has produced a finer-grained sequence of lithostratigraphic units [17,44]. The timescale for this archaeological sequence has been established using a combination of intensive sampling for cosmogenic and paleomagnetic dating [48]. The Earlier Stone Age layers at Wonderwerk Cave yielded a paleomagnetic sequence of Normal (N) and Reverse (R) signatures, from top to bottom of N1/R1/N2/R2/N3 (Fig 1 and Table 1). The upper sample analyzed here is derived from St. 10, which is at the base of the paleomagnetic normal N1 that corresponds to the early Brunhes or Jaramillo [8,48]. The lower sample is derived from the base of St. 11, which is within R2, corresponds to the interval between the Olduvai and Jaramillo subchrons (i.e., 1.79–1.07 Ma). The lower sample is from the base of R2 and an age near the beginning of this interval (ca. 1.8 Ma) is in agreement with the cosmogenic burial ages, the Oldowan-Acheulian lithic artefact transition, and biochronology of the fauna recovered from these layers [46,47,49].

**Table 1:** Context of assemblages analyzed in this study.

Stratum	Lithostrat. Unit	PaleomagZone	Chron/subChron	Age (Ma)	Lithic industry	Depth
St. 10	4a	N1	Early Brunhes or Jaramillo	0.75 or 1.0	Systematic handaxe production	96.60
St. 11	7	Base of R2	Reversal between Jaramillo and Olduvai	1.79-1.07	Initial Acheulean	97.40

Based on color and texture, Beaumont proposed that a white deposit in St. 12 could be an early hearth [46], however subsequent micromorphological analysis demonstrated that this feature was not related to ash or burning [17]. Our research to date has demonstrated extensive evidence for burning in Wonderwerk Cave has been documented in St. 10 in Excavation 1. Micromorphological analysis identified traces of wood ash in this layer along with burning of *in situ* large mammal bone fragments and sediment, both identified by FTIR as burnt, as well as lithic artefacts recovered during excavation with potlid fractures characteristic of heating [8].

The site also provides abundant micromammalian fossils (<1 kg in weight) [49] with signs of digestion indicating that they were the result of predation [18,35] (predominantly from the barn owl *Tyto alba*). Predation was proven in all strata (St. 12-St. 6) and bones ejected by raptors inside pellets that decayed *in situ*. These pellets are ubiquitous in the cave and covered the cave floor like a dense carpet [18,35].

Taphonomic studies of micromammal fossils recovered from Strata 10 and 11, documented changes in texture (e.g., shrinkage, cracking, exfoliation) and color. The incidence of burning in these remains was detected using Backscattered mode of detectors in Scanning Electron Microscopy together with Energy Dispersive Spectroscopy tandem (SEM/BSE-EDS) and Raman analyses to corroborate the results of combustion (carbonization) versus diagenesis (manganese staining) of micromammal bones [35,36,37]. However, while SEM/BSE-EDS has the advantage of being non-destructive, when compared to FTIR, it currently does not allow us to rapidly process a large number of samples since apart from being an expensive method, SEMs are unavailable in field labs. All these limitations prompted the development of a new method for the evaluation of burning on bones.

**Fig 2:**
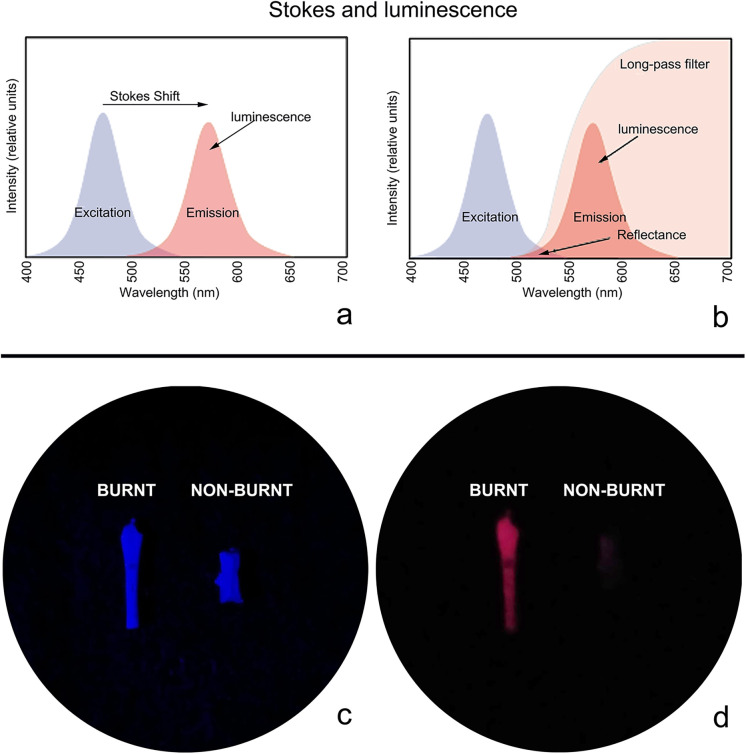
(a) Scheme of luminescence and Stokes shift produced by short wavelength light excitation and luminescent emission. **(b)** Scheme of the area restricted by the long-pass filter. **(c)** Image recovered from the microscope when irradiating fossils with ALS blue light (455 nm) **(d)** same image when observing them with a long-wave pass filter (cut-off 580 nm) yielding a reddish image of the burnt fossils and disappearance of the unburnt fossil.

Consequently, we developed a new, non-invasive analytical method based on luminescence properties of burnt bone. The protocol, adapted from forensic procedures [34], uses an Alternate Light Source (ALS) system to illuminate specimens across a range of wavelengths, with observation through long-pass optical filters (Fig 2, see also Methods section), with which to verify the presence of burning on the micromammal remains.

## Results

In this study we aim to examine how luminescence complements and augments FTIR in the identification of burnt bones. To this end, we have revisited small mammal fossils recovered from Beaumont’s excavations, which had already been studied taphonomically [35,36]. Our research at Wonderwerk Cave followed a stepped workflow to reach a comprehensive understanding of burning on micromammalian fossils. They were first classified by color and tentatively identified as burnt or unburnt [35], prior to applying both FTIR and luminescence (Table 2). Thus, white and grey fossils represent potentially calcined bones, and black and brown represent charred bones, as opposed to unburnt specimens, characterized by whitish-beige or yellowish colors. Our results show that, of the 80 small mammal black and brown bones examined from St. 10 using FTIR analyses, a total of 52 were identified as burnt (positive result) and 28 as unburnt (negative result). Many of these black-brown fossils were included to test the FTIR threshold and its response signal to manganese oxide deposits. For St. 11, the large number of black and brown fossils was unnecessary and we only analyzed 10 black and brown bones, 4 of which were identified as burnt and 6 as unburnt. As mentioned above, one limitation of this result is that it does not allow us to detect black bones that have been burnt below 537º C [39], the temperature at which burning is registered using FTIR. For the black and brown bones, FTIR does however, effectively distinguish burning from discoloration due to diagenetic effects (mainly manganese staining) occurring during fossilization [39,40].

**Table 2:** FTIR and luminescence results for fossil bones analyzed from Strata 11 and 10 at Wonderwerk Cave. Note: positive = indication of burning vs. negative = no signs of burning; brown-black fossils are shown in parentheses.

			FTIR		Luminescence (Binocular human eye)	
	Color	*n*	*p*ositive	negative	positive	negative
**Wonderwerk St. 10**	White	21	10	11	7	14
	Grey	18	16	2	14	4
	**Sub total**	**39**	**26**	**13**	**21**	**18**
	*(Brown-Black)*	*(80)*	*(52)*	*(28)*	–	–
	**WW. St. 10**	**119**	**78**	**41**		
**Wonderwerk St. 11**	White	18	18	0	18	0
	Grey	14	14	0	14	0
	***Sub-total***	***32***	***32***	***0***	***32***	***0***
	*(Brown-Black)*	*(10)*	*(4)*	*(6)*	*–*	*–*
	***WW. St. 11***	***42***	***36***	***6***	***32***	***0***
***Forat de la Conqueta***	*White*	*3*	*3*	*0*	*3*	*0*
	*Grey*	*9*	*9*	*0*	*9*	*0*
	***Sub-total***	***12***	***12***	***0***	***12***	***0***
	*(Brown-Black)*	*(1)*	*(0)*	*(1)*	*–*	*–*
	***Forat Conq.***	***13***	***12***	***1***	***12***	***0***
***Burnt Experimentally***	*White*	*6*	*6*	*0*	*6*	*0*
	*Grey*	*6*	*6*	*0*	*6*	*0*
	***Sub-total***	***12***	***12***	***0***	***12***	***0***
	*(Brown-Black)*	*(1)*	*(0)*	*(1)*	*–*	*–*
	***Burnt exp.***	***13***	***12***	***1***	***12***	***0***
***TOTAL analyzed)***		***187***	***138***	***49***	***77***	***18***
***White and grey bones***		*95*	*82*	*13*	*77*	*18*
***Brown-black bones***		*92*	*56*	*36*	*–*	*–*

The second step of our analysis focused exclusively on the white and grey bones, and it is here that we applied the new methodology based on luminescence properties of burnt bones [34,50,51]. Luminescence consists of a substance absorbing short-wavelength light (near ultraviolet) and emitting it at a longer wavelength. The gap between the absorption and emission peaks is known as Stokes shift (Fig 2a and 2b). This new method involves illuminating the white and grey fossils using a high energy light concentrated in a specific narrow bandwidth (ALS, blue light 455 nm wavelength; Fig 2c). The light emission from the burnt bones can only be seen using a special type of optical filter (named a “long-pass filter”) that blocks shorter wavelengths and allows the long wavelengths of light to be observed. In our case, using the red filter that cuts off at 580 nm, the burnt bones glowed red (Fig 2d), while the unburnt bones did not emit any light.

As expected, no luminescence was detected in any of the 92 blackened specimens analyzed (Table 2), irrespective of whether different ALS lights and filters were applied, as black objects absorb all radiation.

**Fig 3:**
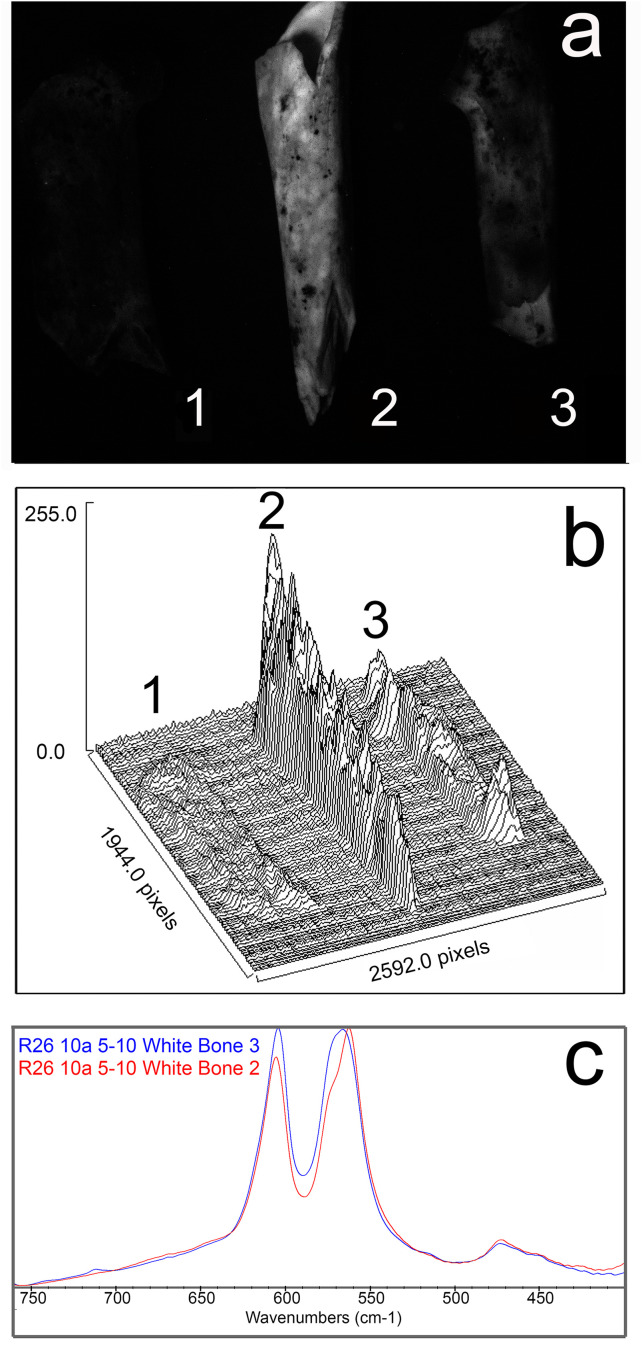
(a) The upper picture shows black and white luminescence images of samples illuminated with a blue light lamp (455 nm) filtered with a long-wave pass filter (530 nm): (1) Unburnt fossil bone (2) burnt fossil bone and (3) fluoridated bone (diagenetic effects during fossilisation) all of them from Wonderwerk Cave site (see Methods section). **(b)** Graph of spectral emission of the same samples: Their spectral content is highly different, especially prominent for the burnt fossil bone (2), lower in the fluoridated fossil bone (3) and the lowest luminescence is provided by the unburnt fossil bone (1). **(c)** FTIR spectra of fluoridated vs non-fluoridated bones. Bone is fluoridated by the relative height ratios between the peak at ~565 cm-1 and 605 cm-1. If the 565 peak is taller than the 605 peak, the bone is not fluoridated (red spectrum R26 10a 5–10 White Bone 2). If the 605 peak is taller than the 565 peak, the bone is fluoridated (Blue spectrum R26 10a 5–10 White Bone 3).

For the 32 white and grey fossil bones from St. 11 at Wonderwerk Cave, both FTIR and luminescence (Table 2) were applied and all of them were identified as burnt by both methods. Both methods identified 21 of the 39 white/grey St. 10 specimens as burnt. Eighteen of the St. 10 white/grey fossils did not show any luminescence. Of these, thirteen bones were not identified as burnt by FTIR, and eight were shown to have been altered by high crystallinity or fluoridation [40]. Thus, luminescence appears to be more sensitive to diagenesis (fluoridation) than FTIR and offers a method which avoids false positives (as can occur with FTIR). We have also observed that the human eye detects luminescence differently than photographic images do, because of their different sensitivity. Using a reflex camera with black-and-white film and illuminating the object with the same blue ALS light (455 nm) but using an orange long-wave filter (530 nm), fluoridated bones can be distinguished as glowing much less than burnt bones. In other words, they show an extremely low luminescence intensity (Fig 3).

In this study we have confirmed that luminescence complements FTIR and distinguishes between burning and diagenetic chemical changes occurring during fossilization as well as, or better than, FTIR.

To further test these results a sample of microfauna from the Bronze Age-Chalcolithic site of Forat de la Conqueta (Lleida, Spain) [52,53] was tested using FTIR and luminescence methods. Burning was detected in 12 white and grey fossils, validated by both luminescence and FTIR. Similarly, for the modern experimental bones burnt by us at temperatures above 700 ºC [54] and characterized by calcination in oxidizing conditions (expressed in their white color), our protocol confirmed burning by showing that these bones were reactive to luminescence.

## Discussion

**Fig 4:**
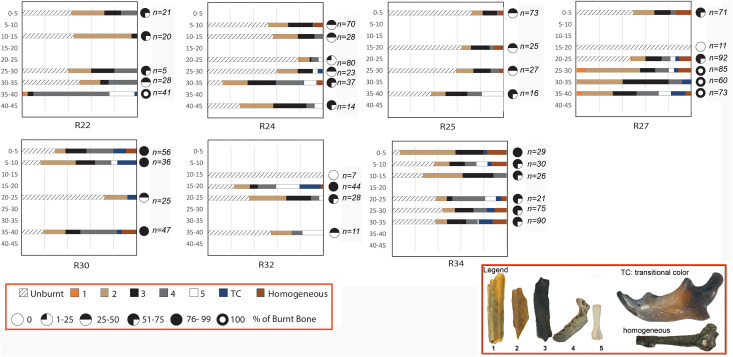
Percentage of burnt bones based on color. The relationship between color and burning in this context is now confirmed by luminescence and FTIR in St. 11. The squares represent the excavation grids (see Fig 1c). Sediment was removed by spits every 5 cm, and small mammals were recovered by wet and dry sieving by Beaumont during excavation (n on the right side of these squares indicate the number of fossils –long bones and jaws- taphonomically characterized as burnt). The legend shows the criteria used in the taphonomic traits, 1 to 5 grades of burning according to color, the transitional color recorded in a unique specimen, and homogeneous black color that appears underneath stains of Mn (all specimens shown in the legend have been proved to be burnt by FTIR).

Our results demonstrate that the combination of luminescence and FTIR is a robust test to assess the presence of combustion and that either method, or both combined, confirm if fossils and modern bones are burnt. Many fossils identified as burnt in the taphonomic study by Fernández-Jalvo and Avery [35] have been re-analyzed and confirmed here. We also corroborate the presence in St. 10 of diagenetic changes to bones caused by fluoridation and fossil diagenesis (Fig 3). In contrast, St. 11 has shown no incidence of diagenetic changes, rather it exhibited consistent evidence of calcination (white-grey color) indicating intense burning. This supports the argument presented in Fernandez-Jalvo and Avery [35] for a discontinuous distribution of burnt bone in St.11 as shown in Fig 4. These authors identified two squares  - R22 and R27  - with only burnt bones, i.e., brown/black and grey/white in color, in contrast to the others that contained both burnt and unburnt bones (Fig 4). Notably, these squares are five meters apart (see Fig 1c). The two bifaces recovered from St. 11 are from squares R25 (spit 5–10) and R30 (spit 30–35).

The long and continuous occupation of the cave by owls [18] would have produced copious amounts of pellets covering the cave floor. The inflammable components of owl pellets (hair, feathers and indigestible prey tissues) would have favored the combustion and/or endurance of a conflagration following the introduction of fire into the cave by hominins. These pellet-associated fires would have left distinct concentrations of scorched microfauna over the cave floor. Since these micromammal remains did not serve a dietary function for hominins, they offer an “independent” data set for assessing the presence of fire in the cave, and clusters of owl pellets on the floor of the cave were burnt following the introduction of fire by hominins.

It is important to note that during the Early Acheulean, Excavation 1 would have been situated ~30 m from the cave entrance [43], such that the evidence for fire is derived from contexts that would have been too deep inside the cave to be affected by natural wildfires. Moreover, the assemblages studied here derive from two distinct and discrete depositional contexts, separated in depth by over 80 cm, and temporally by tens of thousands of years. The numbers of identified micromammals drop significantly in the intervening layer: from an MNI of 5673 in the top of St. 10 to an MNI of 527 in the lowermost part of this stratum, before increasing slightly to 1765 in St. 11, while there are marked differences between strata in Shannon diversity values (H) based on MNIs for the supra-generic groups [49]. These trends corroborate that our samples derive from distinct depositional contexts.

Much research effort to date has focused on identifying the earliest association of hominins with fire since this is especially relevant as a prelude to the cooking food and the provision of additional nutritional value linked to brain enlargement in hominins [20–26]. Wonderwerk has not yielded any evidence for cooking. Rather, the evidence is consistent with opportunistic hominin use of fire, and that early hominins were not passive recipients of natural fires. They introduced fire—probably acquired from wildfires outside the cave—into the shelter and maintained it until it burnt out. As investigations into early pyrotechnology advance, it is increasingly evident that we require robust, non-invasive analytical methods capable of confidently identifying burnt fossils, such as the luminescence method outlined here.

We adapted forensic methodology using diverse ALS lights and filters and demonstrate its effectiveness in an archaeological context. The forensic experiments undertaken by Krap et al. [34] obtained their best results by combining ALS blue lights (peak at 445 nm) and yellow long-wave pass filters (cut-off 476 nm), which enabled the visualization of luminescence. Our best results were also obtained with an ALS blue light, but with a major emission peak at 455 nm for irradiation and a red long-wave pass filter (cut-off 580 nm) for observation. These slight differences between experimentally burnt forensic bones compared with our burnt fossils are likely caused by the loss of organic matter in bone components during fossilization processes, and the fact that we did not use bone cross-sections, but directly analyzed complete fossils to observe luminescence signal. Recently, Krap et al. [55] published another paper detailing the optical property of thermally altered bones: long-decay phosphorescence after UV lighting. This property can indicate the temperatures at which the bones were exposed to heat, which is valuable information for forensic anthropological investigations. However, this method cannot be applied to fossils because these exhibit natural long-decay phosphorescence when exposed to UV light, whether they are burnt or not (see Supporting Information S1 Video). After several tests, the phosphorescence decay time was estimated at 2:09 s (2 seconds and 9 tenths).

One of the most interesting results obtained by us is that, using both methods (luminescence and FTIR), we have confirmed, in a much larger sample, the presence of fire in Early Acheulean St. 10 at Wonderwerk Cave, as already described by Berna et al. [8]. Here we also present new data on burning in St. 11 (with a 100% match between both methods and calcination taphonomic traits) that confirm that burning appears not to be uniform but most intense in discrete areas.. Previously, it was demonstrated that in St. 10, large mammal fossil fragments were burnt and associated with burnt sediments and heat-damaged lithic tools [8]. Our findings are especially relevant for studying a context in which it is difficult to assess the presence of combustion areas [19]. We can now confirm evidence for repeated episodes of burning in the same context (both St. 10 and St. 11, as well as in the distribution of burnt remains within St. 11).

## Conclusions

The burnt fossil bones of small mammals recovered from the early Acheulean deposits in Strata 10 and 11 of Wonderwerk Cave provide strong evidence for repeated, spatially patterned combustion events deep within the cave. The location of these fossils—situated at least 30 m from the cave entrance at the time fossils accumulated—excludes the likelihood that their thermal alteration resulted from the penetration of natural wildfire into the cave. Instead, the recurrence of burning in distinct stratigraphic layers supports the introduction of fire into the cave by hominins. Most notably, fossils from St. 11 are recorded in a paleomagnetic reversal period (R2, Fig 1d) close to the limit with St. 12, which shows normal polarity (N) that could correspond with the beginning of the Olduvai Subchron (1.79 Ma). The intensity of burning is not uniform across the excavated area supporting the argument for multiple burning episodes. Our findings push definitively back the appearance of fire associated with hominins to an age between 1.07–1.79 Ma and confirm evidence of burnt microfauna at St. 11.

The burnt state of these fossils is robustly confirmed through two independent analytical approaches—luminescence and FTIR spectroscopy—providing convergent evidence for in situ heating. Beyond their spatial separation from the cave entrance, the micromammal remains (as part of the cave floor sediment) occur in clear stratigraphic association with Acheulean lithic artefacts and macrofaunal remains, strengthening the link between combustion features and hominin activity. The repetition of this thermal signature across space and time, combined with the broader archaeological context, offers compelling support for intentional fire introduction and use by early Acheulean hominins, most likely *Homo erectus*, on more than one occasion.

More broadly, these findings underscore the value of rigorous, replicable methods for identifying early traces of fire. The new non-invasive optical approach described here, based on the luminescence properties of burnt fossil bone, allows large assemblages of specimens of varying sizes to be assessed rapidly and without destructive sampling. This methodological advance provides a powerful tool for identifying heating events and mapping their spatial distribution within archaeological sites. As applied to Wonderwerk Cave and future contexts, it offers the potential to refine our understanding of the emergence of hominin pyrotechnology, its behavioral significance, and its ecological underpinnings during the early Acheulean.

## Materials

Wonderwerk Cave has provided abundant micromammal remains with signs of digestion indicating that they were result of predation, predominantly by the barn owl (*Tyto alba*) that roosted in the cave [18,35]. For the current study we analyzed a sample of 161 micromammal fossil bones from St. 10 and St. 11 of Wonderwerk. The fossils were randomly selected, and brown/black bones were included despite the fact that we identified Mn oxides that blackened some of the fossil surfaces. These fossils were included in the analysis with the intention of distinguishing Mn staining from burning, and to test the FTIR spectrometry response and its threshold temperature. These brown/black bones, however, cannot provide any kind of luminescence.

In addition to the Wonderwerk micromammals, 13 fossil bones from a Chalcolithic-Bronze Age European site, Forat de la Conqueta, (Lleida, Spain) [52]^,^ a rock shelter with evidence of ceremonial burning [53], were tested using the same methods as were 13 calcined, modern large and small mammal bones that had been experimentally burnt by us [36,54] in a wood pyre and muffle furnace at high temperatures (above 750 ºC). A total of 187 bones were examined in this study (Table 2). This is one of the largest samples of fossils ever analyzed by FTIR, despite that FTIR analytical procedure requires the analysis of individual samples. Larger numbers of fossils can be examined using optical methods, such as luminescence, when illuminated simultaneously with appropriate ALS light (455 nm) and when the red long-wave filter is adjusted to the microscope eyepiece or worn in glasses (forensic goggles).

## Analytical protocols

An important indicator of fire is the presence of burnt bones, which are commonly identified by changes in texture, structure, and color [56,57]. The first step in the analysis of the fossil Wonderwerk bones involved sorting the assemblage based on the macroscopic identification of different colored bones (characterized using an AVANTES AvaSpec-2048FT spectrophotometer) tentatively identified as burnt in oxidizing conditions [58] as described in results. However, combustion color and textures may frequently be mimicked by post-depositional and fossilization processes (e.g., weathering, soil chemistry, or mineral staining [57,59]). For example, in a previous study [8,39] some white-colored fossils from St. 10 were shown (by FTIR) to be fluoridated (i.e., fluoride ions from the surrounding sediments or waters incorporated into the hydroxyapatite mineral structure during diagenesis) rather than calcined.

### *Fourier transform infrared* (*FTIR*)

FTIR spectroscopy is a molecular analytical technique well-suited to identify heat-related transformations in materials of different nature, such as clay minerals and bone [33,39–41], In particular, because of high temperature, the bone mineral —namely carbonate-hydroxyapatite—undergoes characteristic recrystallisation, and through the removal of carbonates, forms pure hydroxyapatite [58]. The recrystallisation occurs at approximately 537 °C [40] and above, and can be discerned via the sharpening of the ν4 PO4 (565 cm^−1^–630 cm^−1^) and ν3 PO4 (1,020 cm^−1^–1,100 cm^−1^) bands in FTIR spectroscopy. The sharpening of peaks caused by high temperature and the removal of carbonates results in the appearance of a characteristic peak at 630 cm^−1^. A peak at 1,096 cm^−1^ does not appear in fresh bone but appears in archaeological and fossil bone due to its higher crystallinity [33,39,40,58,60,61].

Samples of bone were powdered and mixed with 5 mg of KBr. The mixture was pressed into a 7 mm die using a PikeTM hand press and analyzed with a Thermo Nicolet iS5 FTIR spectrometer. Thirty-two scans per sample were collected at a resolution of 4 cm^-1^. FTIR spectra were analyzed using Omnic software.

The presence of the FTIR 630 cm^-1^ hydroxyapatite peak was determined using the HATI method as described in Shaw [39]: a baseline was set between the limits of 655 cm^-1^ and 625 cm^-1^. The area under the spectrum and above the baseline was measured between these parameters. Positive areas indicate bone heated to above 537 °C, while negative areas indicate undetectable hydroxyapatite in the sample, and therefore undetected heating using this method. Fluoridation of bone was determined through the relative intensities of the absorption peaks at 606 cm^-1^ and 567 cm^-1^ [37, Fig 3c].

### Scanning Electron Microscopy-Energy Dispersive Spectroscopy tandem (SEM-EDS tandem)

**Fig 5:**
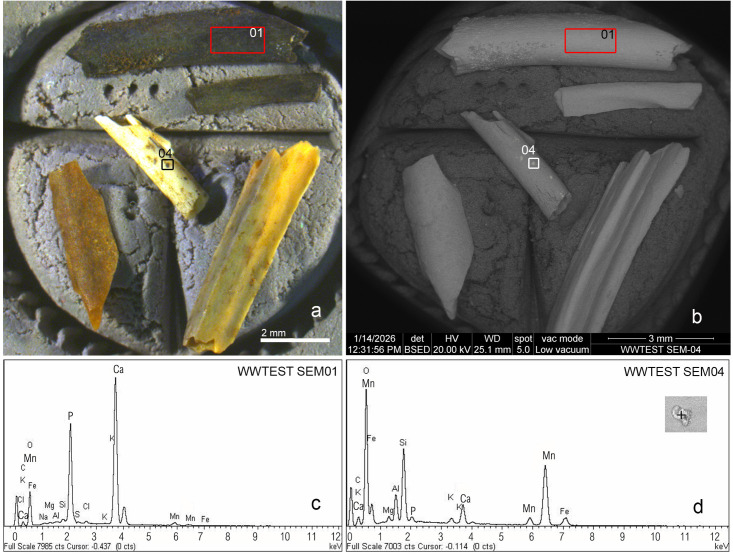
SEM-EDS test [35,37] of burnt fossils here validated by FTIR. **a)** Optical image showing different degrees of cremation (clockwise from the top: two charred remains (cremation grade 3) with a uniform black color; bottom right: a slightly burnt remains at grade 1 with dispersed brownish cremation spots; left: a grade 2 brown burnt bone remain; center: a calcined fossil –grade 5-, with dispersed black manganese oxide spots). **b)** The same image taken with SEM using backscattered electron (BSE) detectors (note the manganese spots in the calcined fossil in the center appear brighter when viewed in SEM-BSE mode). **c)** EDS spectrum of the area marked with a red rectangle 01. If the blackish color of these fossils were the result of manganese oxides, the spectrum would show a strong peak for this element. **d)** Spectrum of manganese oxide spot (04).

This methodology, which was first proposed in Wonderwerk [37], was also applied to fossils from other sites [38] to distinguish dark charred bones from those stained by manganese oxides (Fig 5a). Images from a scanning electron microscope using backscattered electron detector (BSE) together with energy dispersive X-ray spectroscopy (EDS) enables distinguishing between these two types of bones. Backscattered electrons reveal differences in sample density. Thus, mineralized areas (e.g., manganese coatings) appear brighter and whiter at the SEM since manganese is metallic in nature. In contrast, charred bones show a homogeneous grey color at the BSE mode since the calcium phosphate composition of bone is less metallic than manganese (Fig 5b). The EDS spectrum shows only the bone’s calcium phosphate composition (Fig 5c), confirming that the black color is due to combustion (grade 3 carbonization). EDS easily detects the presence of manganese (Fig 5d) and shows a peak for this element when the black color is due to manganese oxide staining. In the case of the samples in Fig 5, FTIR revealed that they were all burnt, and the bones appeared grey in BSE mode. EDS analysis of the black bones shows only calcium phosphate. SEM-BSE-EDS tandem only allows simultaneous analysis of few fragments or small mammal bones, reducing the effectivity of this spectrometry in microfaunal assemblages that usually yield several hundreds of specimens.

### Luminescence

Luminescence entails the emission of light that does not come from the temperature of the emitting body or its reflection. It is caused by chemical, biochemical, or crystallographic changes, the motions of subatomic particles, or radiation-induced excitation of an atomic system.

Photoluminescence is a type of luminescence, in which two processes are differentiated based on the delay between absorption and emission of light: fluorescence (very short delay, around 10^−6^–10^−8^ s) or phosphorescence (delay 10^−6^ s and longer, see S1 Video). Sometimes, as seen here, a substance excited by light emits light in another wavelength (Stokes shift), which can be optically detected using long-pass filters. Lambrecht and Mallol [62] used bone auto-fluorescence of modern bovine cortical bone sections burnt in a muffle oven, applying blue light (400 nm to 440 nm) using an epifluorescent microscope. The research undertaken by them resulted in an important finding that facilitates detection of burning below 300 °C– 400 °C in carbonized (black-brown) bones, i.e., below the current threshold of FTIR spectroscopy [39,58]. However, their findings were based on experimentally heated bones, so their protocol has to be tested in fossils that have manganese coating, discoloration, and diagenetic processes that may distort or absorb the weak auto-fluorescence signal of carbonized fossil bones. On the other hand, their observations were obtained from prepared cut-section samples, and thus involve an invasive technique that hampers the examination of large numbers of fossil specimens, while the epifluorescence microscope they used cannot be taken into the field. Despite this, their results increase multi-proxy data available to evaluate the heating temperature, especially at incipient stages of burning.

Our protocol uses Alternate Light Sources, an approach that has been extensively used in forensic investigations to analyze luminescence properties of fluids and skeletal remains [34,50,51,55]. These devices provide narrow bandwidth radiation in the short visible (NBV) and ultraviolet (UV) spectra [63,64]. We adapted the forensic protocol to our specimens. To prevent interference with light of other wavelengths, the luminescence observations were optimal when the samples were placed in a dark chamber. This consisted of illuminating the bone specimen with a narrow band wavelength light (blue ALS light, 455 nm wavelength; Fig 2a) which emits light at a longer wavelength that will only be visible using a filter (named a “long-pass filter”), in this case a red long-pass filter that cut-off at 580 nm (Fig 2b–2d).

To evaluate the reliability of our results, blind tests were undertaken by seven independent research participants in our laboratory plus three of the co-authors (DMM, SGM, YFJ). Luminescence was described by all ten observers as reddish in appearance when seen with the red long-wave pass filter (Fig 2d), while bones that were not burnt or altered by diagenesis disappeared (Table 2).

**Fig 6:**
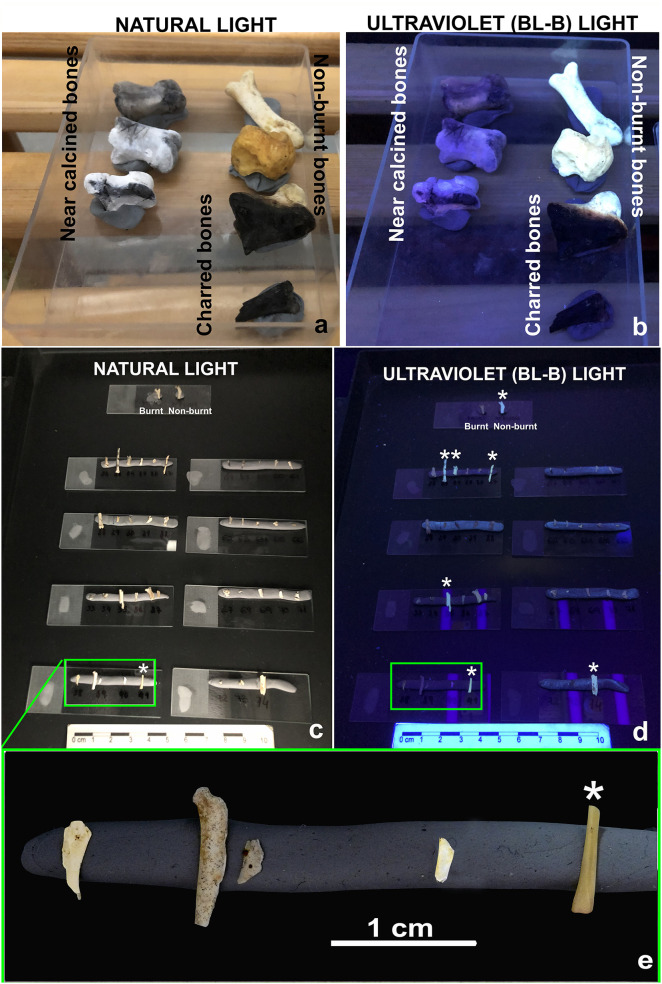
(a) Picture taken under natural light of burnt modern experimental phalanges. **(b)** Picture taken under black-light blue ultraviolet (BL-B) light of burnt modern experimental phalanges. **(c)** Picture taken under natural light of burnt and non-burnt small mammal fossils from Wonderwerk Cave. **(d)** Picture taken under ultraviolet (BL-B) light: burnt fossil bones become obscure and dark purple while non-burnt fossils glow brightly (asterisks). **(e)** Detail of bottom left slide (green rectangle). Fossil on the right hand of the slide (asterisk) has a yellowish color that indicates the fossil is not burnt.

Our aim is that any researcher can use this method, but in the course of sorting by color, it became challenging for untrained researchers to reliably distinguish between non-burnt (natural color, i.e., whitish-beige or yellowish) and white calcined/fluoridated fossils. Seven out of ten volunteers were paleontologist taxonomists and failed in trials when they were asked to distinguish between natural color and potentially burnt bones. To address this difficulty, we developed a pre-analytic procedure consisting of illuminating the samples with black-light blue ultraviolet lamps (BL-B UV, peak at 368 nm) which are commonly used for stage illumination, discos or adverts. Under black BL-B UV light, unburnt bones glowed brightly, contrasting with calcined bones (grey/white), which appeared obscure and dark purple (Fig 6 and Table 3).

**Table 3:** Summary of the procedure followed in this paper according to fluorescence and luminescence properties of fossil and bone samples.

Summary	Material	Fluorescence	Luminescence
		BL-B UV (368 nm)	Blue light +filter
**BURNT** **Calcined**	FOSSIL BONE	NONE	455 nm + red filter [this paper]
	EXPERIMENTAL BONE	NONE	445 nm+yellow filter [46]
**UNBURNT**	FOSSIL BONE	YES	NONE
	EXPERIMENTAL BONE	YES	NONE
**DIAGENESIS** (fluoridation)	FOSSIL BONE	NONE	NONE or very weak

This finding corroborates an optical property predicted by Lambrech and Mallol [50] that a “stronger difference in fluorescence between unheated and calcined bone under UV-lights would be expected” (their page 11), and a recent publication by Thompson and colleagues [51] reinforced this finding. Therefore, we compared a photograph taken under natural light of the white fossils (Fig 6c) with a photograph taken under BL-B UV (Fig 6d), and sent it to 25 researchers, archaeologists, taphonomists and taxonomists. All 25 could successfully discriminate the extremely bright, natural non-burnt fossils (Fig 6c-asterisks), from darkened purple fossils that included both burnt and diagenetic fluoridated fossils.

Based on UV light alone, none of the volunteers could distinguish between the burnt and diagenetically altered fossils of St. 10. Only by using our protocol with the appropriate blue ALS (455 nm wavelength) and red long-pass filters could the calcined fossils be differentiated from fluoridated whitened fossils affected by diagenesis (as seen in Fig 3). This protocol can be applied in other sites and contexts [9–11,14,27,28,65], including on site. Combining our protocol with those of forensic field searches [51] allows for the detection of the spatial distribution of calcined fossils on an open excavation surface in the dark or at nighttime using red long-pass filter glasses (forensic goggles) lighting the surface with a handheld blue ALS (455 nm).

In summary, luminescence analysis offers distinct advantages: it is rapid, non-invasive, scalable to large sample sets, and supported by equipment that is inexpensive, portable, and fully field-deployable. This positions luminescence as a transformative tool for future research on early fire use and its role in hominin evolution.

### Photographic method to record luminescence

Luminescence has been tested in modern experimentally and forensic burnt bones, but diagenesis compromises positive results in fossils and requires further research and experiments.

**Fig 7:**
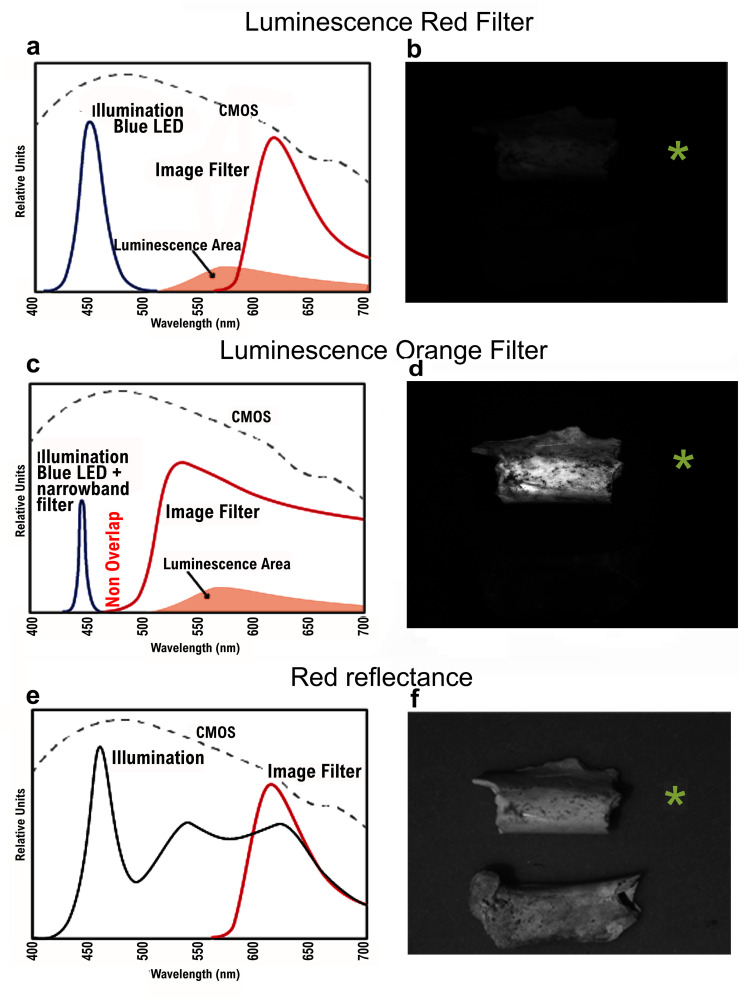
Spectral distributions of elements used in the luminescence phenomenon described here: Images of a burnt fossil (always on top) and an unburnt fossil, both classified correctly by FTIR. **(a)** Scheme when using the red long-wave pass filter (cut-off 580 nm) and **(b)** Photograph taken using the red long-wave pass filter showing a too dark image that could not show this luminescence phenomenon as clearly as the human eye can detect. **(c)** Spectro-radiometer measurements taken by adding a narrowband filter to the blue light to prevent any possible overlapping between irradiation and observation spectra; **(d)** Irradiation with a blue light (455 nm) observed with an orange long-wave pass filter (cut-off 530 nm) luminescence were used to take the pictures (camera sensitivity shown as CMOS) of Fig 3; **(e)** Scheme when using natural white LED light (LED D50 full spectrum within the visible) and long-wave pass red filter **(f)** Picture of burnt (top) and unburnt (bottom confirmed by FTIR) bones that show a similar image.

The combination used to identify luminescence in fossils under the microscope by a human observer, differs from the long-pass filter with photographic equipment that enhances the luminescence using the orange camera filter (cut off 530 nm). This setup provides better quality image because of the higher luminous signal, allowing more accurate documentation of the phenomenon (Fig 7a–7d) and may distinguish fluoridated bones (Fig 3).

A DMK 72AUC02 reflex camera from manufacturer The Image Source, equipped with an Aptina MT9P031 monochrome sensor, was used for graphic recording. Astronomik L-RGB Typ 2c filters that comply with DIN 5032 were used as the R, G, and B filters for reflectance testing in the R, G, and B bands. An Astrodon UV Venus filter was used for UV reflectance testing.

Luminescence preparation: Burnt and non-burnt fossil bones were excited with a Megamaxx™ Alternate Light Source (ALS) System (Sirchie, Youngsville, NC, USA). The Alternate Light Sources (ALS) used in our experiments were: 355 nm (UV), 368 nm (BL-B UV), 410 nm (violet), 455 nm (royal-blue), 470 nm (blue), 505 nm (cyan), 530 nm (green), 590 nm (orange), and 625 nm (red). These lights were used in combination with three Tiffen photographic filters that act as long- wave pass filters blocking emission of shorter wavelengths (from yellow 476 nm, orange 530 nm to red 580 nm).

Specimens were illuminated with the ALS (455 nm) mounted on a tripod and the long-wave pass filter was attached to the base of the objective (between the specimen and the objective). The observations were conducted in complete darkness (dark room) to prevent external illumination.

Luminescence was recorded by a spectroradiometer hosted at the Optics Institute “Daza de Valdés” (CSIC), adding an interference filter centered at 450 nm with a 10 nm bandwidth (Fig 7c), to check that other unwanted irradiation spectral bands from the lamp were blocked. The spectroradiometer used is a large equipment Minolta CS-1000. In order to recreate the same capture conditions as with the CMOS camera, we used the same blue light source (455 nm) and an interference filter at 450 nm, together with the Tiffen orange long wave pass filter. In this way, the spectroradiometer clearly shows a difference between the luminescence of the burnt bone compared to the non-burnt fossil sample (Fig 3).

In order to distinguish between luminescence and reflection phenomena, two types of experiments were performed. The first one involved daylight (LED D50 full spectrum within the visible) combined with red (R) and blue (B) Astronomik filters (Fig 7d and 7e). The second experiment, conducted with UV light and UV filter (Astrodon), is not shown in Fig 7, but yielded a similar result. These experiments showed that both specimens had a similar reflectance (Fig 7f), confirming that the intensity variation collected in the image originates only from luminescence, and never from a reflection resulting from overlapping the excitation light with the band of the supposed emission limited by the filter.

Significance statement

Identifying the earliest use of fire is critical for understanding a cornerstone innovation in human evolution. Yet evidence of burning in deep-time archaeological contexts is notoriously difficult to detect and evaluate. This study provides some of the earliest, securely contextualized traces of fire use by hominins and introduces a rapid, non-invasive method for identifying burnt bone at high temperatures for investigating when and how hominins first began to engage with fire.

## Supporting information

S1 Video SI-1 Video of Wonderwerk fossils displaying the phosphorescence after UV lighting, whether they are burnt or not. Decay time has been estimated at ~2 seconds (see Main Text).
(MP4)
